# Protective effects of medicinal plant-derived metabolites in cardiovascular disease targeting exosomal pathways

**DOI:** 10.3389/fphar.2025.1681625

**Published:** 2025-10-27

**Authors:** Liwen Fu, Jiaxiu Han, Rong Lu, Aifeng Song, Ao Shen, Changjiang Xiong, Yanzhu Liu, Zu Gao

**Affiliations:** ^1^ Innovative Institute of Chinese Medicine and Pharmacy, Shandong University of Traditional Chinese Medicine, Jinan, China; ^2^ Rehabilitation Medical College, Shandong University of Traditional Chinese Medicine, Jinan, China; ^3^ International Education College, Shandong University of Traditional Chinese Medicine Jinan, Jinan, China; ^4^ ACUPUNCTURE & WELLNESS, New York, NY, United States; ^5^ College of Traditional Chinese Medicine, Shandong University of Traditional Chinese Medicine, Jinan, China

**Keywords:** cardiovascular disease, exosomes, miRNA, medicinal plant-derived metabolites, pharmacological effects

## Abstract

Cardiovascular disease (CVD) is a highly prevalent and lethal disease worldwide, and existing conventional therapeutic drugs have limitations due to their side effects. Medicinal plant-derived metabolites have become a research hotspot due to their multi-target and multi-pathway cardioprotective potentials, while exosomal miRNAs, as core regulatory molecules of intercellular communication, play a key role in CVD such as atherosclerosis and myocardial infarction. This paper systematically reviews the mechanisms by which medicinal plant-derived metabolites regulate exosome miRNA or synergize with exosome therapy to protect CVD. For instance, Tanshinone IIA, Astragaloside IV, Paeonol, and Ginsenoside Rg1 can achieve effects such as promoting/inhibiting angiogenesis, anti-inflammation, and vascular remodeling by regulating exosomal miRNA expression. Finally, we look forward to the future direction of Medicinal plant-derived metabolites combined with exosomes in the protection of cardiovascular diseases, and provide a theoretical basis for the application of Medicinal plant-derived metabolites in CVD protection and the development of exosomal miRNA-targeted drugs.

## 1 Introduction

Cardiovascular disease (CVD) is one of the leading causes of morbidity and mortality worldwide, affecting more than 500 million people globally and causing 18.6 million deaths annually (Inam et al., 2023; Vaduganathan et al., 2022), making it a major global health issue. Statins, angiotensin-converting enzyme inhibitors, angiotensin II receptor blockers, beta-blockers, and other drugs form the foundation of cardiovascular disease treatment (Netala et al., 2024). However, these drugs may tigger side effects such as gastrointestinal reactions, electrolyte disturbances, kidney damage, dizziness, etc. (Khatiwada and Hong, 2024; Ko et al., 2002; Chen et al., 2021). In addition to the conventional therapies mentioned above, herbal medicine has attracted considerable attention for its potential cardiovascular benefits (Hao et al., 2017). Active ingredients in natural products, such as terpenoids, polysaccharides, and phenolic compounds, have demonstrated good cardioprotective effects and help enhance cardiovascular health (Zhao et al., 2020).

Exosomes (Exos) are cell-derived lipid bilayer microvesicles that can carry various bioactive molecules, such as microRNA (miRNA), soluble proteins, and membrane proteins (Bofill-De Ros and Vang Orom, 2024). As a key mediator of intercellular communication, exosomes transmit biological signals by targeting and recognizing recipient cells and releasing their contents, of which miRNAs are the core molecules mediating this process due to their highly efficient regulatory activities (Macias et al., 2015). miRNAs are a class of endogenous non-coding RNAs with highly conserved sequences that are capable of regulating post-transcriptional gene expression (Lu and Rothenberg, 2018), And the gene network regulated by miRNAs is a complex gene regulatory network involving multiple targets and multiple pathways (Miao et al., 2018). Research has shown that exosomal miRNA participates in the occurrence and development of various cardiovascular diseases, such as atherosclerosis, myocardial infarction, and myocardial ischemia-reperfusion, by regulating the expression of target genes. This deep involvement in the disease process has also led to increasing attention being paid to extracellular research in the field of cardiovascular medicine (Zheng et al., 2021).

Medicinal plant-derived metabolites, especially those derived from herbal medicines, such as terpenoids, polyphenols, polysaccharides, alkaloids, and other compounds, also exhibit multi-pathway and multi-target characteristics when exerting their effects. Numerous studies have shown that these medicinal plant-derived metabolites can modulate exosomal miRNAs, thereby achieving stable cellular phenotypes, anti-inflammatory effects, and enhanced cellular antioxidant capacity in the treatment of cardiovascular diseases (Shi et al., 2020; Fu et al., 2021; Yang et al., 2024). This review will conduct a comprehensive analysis of both *in vivo* and *in vitro* experiments, and summarize the research progress on the medicinal plant-derived metabolites that regulate exosome miRNA expression or cooperate with exosomes in the treatment of CVD.

## 2 Data collection methods

We searched PubMed, Web of Science, and the China National Knowledge Infrastructure (CNKI) database with the keywords “Cardiovascular Disease” “Medicinal plant-derived metabolites” “Exosomes” “miRNA” and their combinations. The search was conducted until 1 July 2025 (no time limit before this date). Inclusion criteria: (1) Original experimental studies (*in vivo* or *in vitro*) investigating the therapeutic effects of single or well-defined components from medicinal plant on CVD; (2) The medicinal plant-derived metabolites is a verifiable bioactive monomer, which exerts its therapeutic effects on CVD through synergistic interaction with exosomes or regulation of exosomal microRNAs; (3) Cell experiments, animal studies or preclinical research directly linked to the pathological mechanisms of CVD; (4) The literature should be in English or Chinese, and the type should be a peer-reviewed journal article or a degree thesis. Exclusion criteria: (1) Medicinal plant are crude extracts or mixtures of unidentified constituents; (2) The focus of the literature review lies in the toxicological effects or pharmacokinetic characteristics of medicinal plant, rather than their pharmacological mechanisms for treating CVD; (3) Research exclusively utilising exosomes as drug delivery vehicles for the treatment of CVD; (4) Medicinal plant-derived metabolites exert their effects not via the exosomal pathway, but by directly regulating intracellular microRNAs or modulating non-exosomal extracellular vesicles; (5) Studies with major flaws in experimental design, such as the absence of a negative control group or insufficient sample size; (6) The paper is included in the conference proceedings but the full text is not available for reading. Through the above search methods and inclusion/exclusion criteria, we initially reviewed the titles and abstracts of the included articles, then carefully read the full texts, ultimately including 12 studies (Table 1).

**TABLE 1:** The medicinal plant-derived metabolites that regulate exosome miRNA expression or cooperate with exosomes in the treatment of CVD and related factors.

Metabolite	Targeted miRNA	Main pathway	Cardiovascular outcome	References
Tanshinone ⅡA	miR-223-5p↓	miR-223-5p/CCR2	LVEF, LVFS, +dP/dt_max, -dP/dt_min, Microvascular density↑; MI area, Inflammatory cell infiltration, Cell apoptosis, Collagen fibers, Macrophage abundance, CCR2 Monocyte↓	Li et al. (2023)
Astragaloside IV	miRNA-411↑	miR-411/HIF-1α	LVEF, LVFS, CD31, VEGF, Tube formation↑; LVEDD, LVESD, Collagen deposition↓	Yang et al. (2023)
Astragalus polysaccharide	--	PI3K/Akt	Vascular grid, Cell migration rate, Survival rate of myocardial cells, PI3K, p-PI3K, Akt↑; Apoptosis rate↓	Guo et al. (2023)
Zedoarondiol	miRNA-let-7a↓	--	THBS-1, CD36↑; Density of new blood vessels, Platelet activation rate, VEGF, ox-LDL, TNF-α, MMP-9↓	Xie et al. (2025)
Paeonol	miRNA-223↑	STAT3	IL-1β, IL-6, VCAM-1, ICAM-1, STAT3, pSTAT3↓	Liu et al. (2018)
Paeonol	miRNA-223↑	NLRP3	RAEC survival rate↑; TC, TG, IL-1β, IL-6, NLRP3, ASC, caspase-1, ICAM-1↓	Shi et al. (2020)
Ginsenoside Rh2	--	HMGB1/NF-κB	Cardiomyocyte homing ability↑; NF-kB p65, NLRP3, HMGB1↓	Qi et al. (2022)
Tanshinone ⅡA	--	PI3K/Akt	Cell vitality, Tube-forming activity, Akt mRNA, PI3K mRNA, p-PI3K, p-Akt↑; LDH leakage rate, ROS, IL-1β, IL-6, TNF-α↓	Ma et al. (2023)
Astragaloside IV	--	PDHA1	LVEF, LVFS, PDHA1↑; LVEDS, LVEDD, IL- 6, TNF-α, MI area, Apoptosis rate of myocardial cells↓	Su et al. (2025)
Ginsenoside Rg1	miRNA-7977↑	miRNA-7977/MAPK13	α-SMA, SM-MHC, Smoothelin-B↑; Proliferative activity, Proliferation index, Smemb, MAPK13↓	Shuai (2022)
Curcumin	miRNA-92b-3p↑	miR-92b-3p/KLF4	KLF4, RUNX2↓	Chen et al. (2022)
Oridonin	--	--	CD63, CD81, AliX, Beclin-1, ATG13, Bcl-2, Ki67, EdU positive cells↑; Cell apoptosis, heart rate, LVSP, LVFS, LVEF, LVWT, Apaf1, Bax↓	Fu et al. (2021)

## 3 Mechanism of action of medicinal plant-derived metabolites in regulating exosomal miRNA therapy for CVD

In the pathological progression of CVD, mechanisms like angiogenesis, inflammation, oxidative stress, vascular remodelling, and autophagy interact complexly. Excessive reactive oxygen species-induced oxidative stress is a pivotal trigger (Taleb et al., 2018): it activates inflammatory pathways to worsen vascular wall inflammation via macrophage infiltration and inflammatory mediator release, and damages vascular endothelial cells to disrupt vascular homeostasis and initiate vascular remodelling (Totoń-Żurańska et al., 2024). The inflammatory response further amplifies oxidative stress and bidirectionally influences angiogenesis by regulating factors such as VEGF and AngⅡ, thus compensating for ischaemic injury (Hu et al., 2025); however, excessive angiogenesis may cause vascular leakage and intra-plaque inflammatory infiltration, exacerbating plaque instability (de Vries and Quax, 2016). During vascular remodelling, inflammation and oxidative stress regulate smooth muscle cell proliferation/migration and extracellular matrix restructuring, while indirectly affecting autophagy by altering vascular architecture (Whiteford et al., 2016; Gallo et al., 2018). As a cellular homeostasis mechanism, autophagy eliminates oxidatively damaged proteins/organelles to suppress inflammation and remodelling, but persistent oxidative stress or severe ischaemia may induce excessive/insufficient autophagy, exacerbating cell apoptosis and tissue damage (Ren et al., 2023). These mechanisms form a multidimensional network, and disrupted dynamic equilibrium thereof is the core pathological basis for CVD onset and progression.

### 3.1 Promote/inhibit angiogenesis

Angiogenesis is the process by which endothelial cells proliferate, differentiate, and migrate to generate new capillaries through budding or anastomosis on the basis of existing microvenules (Gete et al., 2021). Following myocardial ischemia or myocardial infarction, angiogenesis represents a crucial pathway for resolving ischemic injury, restoring myocardial blood supply, and improving cardiac function (Yang et al., 2025; Wu et al., 2021). However, angiogenesis occurring within atherosclerotic plaques significantly compromises plaque stability, leading to intraplaque hemorrhage and triggering plaque rupture (Perrotta et al., 2019; Brezinski et al., 2019).

Tanshinone IIA is a triterpenoid compound with potent protective effects on the cardiovascular system (Qin et al., 2020). Studies have shown that Tanshinone IIA can significantly improve left ventricular ejection fraction (LVEF), left ventricular fractional shortening (LVFS), maximum rate of pressure rise (+dP/dt_max), and minimum rate of pressure decline (-dP/dt_min) in rats following myocardial ischaemia-reperfusion injury in rats by down-regulating miRNA-223-5p derived from bone marrow mesenchymal stem cells. And reduce infarct size and collagen deposition, alleviate inflammatory cell infiltration and apoptosis, and inhibit chemokine (C-C motif) receptor 2 (CCR2) activation, thereby reducing monocyte infiltration, promoting angiogenesis, improving MIRI (Li et al., 2023).

Astragaloside IV is a triterpenoid compound exhibiting biological activities such as antitumor, antioxidant, and anti-inflammatory effects (Yao et al., 2023; Liang et al., 2023). Animal studies have demonstrated that exosome-derived from bone marrow mesenchymal stem cells (BMSC), induced by astragaloside IV via the miR-411/HIF-1α axis, can mitigate myocardial damage caused by AMI, which is primarily manifested by a reduction in left ventricular end-diastolic diameter (LVEDD) and left ventricular end-systolic inner diameter (LVESD), increasing LVEF and LVFS, reducing collagen deposition and CD31 expression in myocardial tissue, and inhibiting the expression of the target gene HIF-1α (Yang et al., 2024).

Astragalus polysaccharides are polysaccharide compounds with immunomodulatory effects, therapeutic benefits for cardiovascular diseases, and antitumor properties (Li et al., 2022; Su et al., 2025; Zhang et al., 2024). Guo et al. demonstrated that Astragalus polysaccharide combined with exosomes derived from adipose-derived mesenchymal stem cells can promote angiogenesis and endothelial cell migration through the PI3K/Akt pathway, increase the survival rate of AC16 cells, reduce myocardial cell apoptosis, and lower the expression levels of Bax and cysteine-dependent aspartate-specific protease-3 (caspase-3). Increase the expression levels of B-cell lymphoma-2 (Bcl-2), phosphatidylinositol 3-kinase (PI3K), phosphorylated phosphatidylinositol 3-kinase (p-PI3K), and protein kinase B (PKB) protein expression levels, thereby achieving a protective effect on damaged myocardial tissue (Guo et al., 2023).

Zedoarondiol is a terpenoid compound exhibiting anti-inflammatory, antiviral, and antioxidant biological activities (Ko et al., 2018; Barcellos Marini et al., 2018). Research has found that Zedoarondiol downregulates the levels of platelet-derived exosomal miRNA-let-7a, thereby increasing the expression of aortic thrombospondin-1 (THBS-1) and cluster of differentiation 36 (cluster of differentiation 36, CD36) expression levels, inhibiting aortic plaque formation, reducing vascular neogenesis within plaques, and lowering the expression levels of vascular VEGF and plasma low-density lipoprotein (LDL). It also reduces the concentration of matrix metalloproteinase-9 (matrix metalloproteinase 9, MMP-9) and tumour necrosis factor-alpha (TNF-α) concentrations, ultimately exerting anti-AS activity (Xie et al., 2025).

### 3.2 Anti-inflammatory

Chronic inflammation is a major risk factor for CVDs (Prousi et al., 2023). It damages vascular endothelial cells, promotes AS plaque formation, and may lead to plaque instability triggering thrombosis, thereby increasing the risk of diseases such as myocardial infarction. Simultaneously, when CVDs like myocardial ischemia occur, they further activate the body’s inflammatory response, creating a vicious cycle (Sagris et al., 2021).

Paeonol is a phenolic compound with pharmacological effects such as anti-inflammatory, anti-tumor and metabolic regulation, and has good application value in the treatment of CVD (Yang and Li, 2022). Research has confirmed that the mechanism by which paeonol inhibits AS may involve upregulating the levels of monocyte-derived exosomal miRNA-223, thereby inhibiting the inflammatory pathway of signal transducer and activator of transcription 3 (STAT3) and its downstream inflammatory factors, including interleukin-1β (IL-1β), interleukin-6 (IL-6), intercellular adhesion molecule-1 (ICAM-1), and vascular cell adhesion molecule-1 (VCAM-1) (Liu et al., 2018). Another studies have shown that paeonol can increase the expression level of plasma exosomal miR-223, inhibit the downstream NLRP3 inflammasome pathway, reduce serum TC, TG, IL-1β and IL-6 levels, improve the survival rate of RAECs, and play an anti-inflammatory role in endothelial cells of hyperlipidemic (HLP) rats (Shi et al., 2020).

Ginsenoside Rh2 is a triterpenoid compound exhibiting multiple pharmacological activities, including antitumor effects, improvement of cardiac function and fibrosis, anti-inflammatory properties, and antibacterial activity (Liu et al., 2022). Qi et al. found through an *in vitro* model of AMI that ginsenoside Rh2 modulates the NF-κB signalling pathway via the high mobility group box 1 (HMGB1)/nuclear factor kappa-light-chain-enhancer of activated B cells (NF-κB) to improve the OGD environment, enhance the homing ability of cardiomyocytes, thereby inhibiting the nuclear translocation of NF-κB p65 and the activation of the NLRP3 inflammasome, and enhancing the protective effect of BMSC-derived exosomes on damaged myocardium, providing new evidence for the regulation of exosomes by traditional Chinese medicine in the treatment of cardiovascular diseases (Qi et al., 2022).

### 3.3 Anti-oxidative stress

Physiological levels of ROS can act as signaling molecules to regulate a wide range of processes in the cardiovascular system and contribute to the maintenance of cardiovascular homeostasis. However, excessive production or persistently elevated ROS levels play a pivotal role in the onset, progression, and clinical outcomes of CVD (Csányi and Miller, 2014).

In the process of AS, vascular endothelial injury serves as the initiating step (Yubero-Serrano et al., 2020). Research has found that Tanshinone ⅡA synergises with mesenchymal stem cell (MSC)-derived exosomes can enhance the therapeutic effect on AS. This is achieved by enhancing the proliferation and tubularisation capacity of thoracic aortic endothelial cells, upregulating the mRNA and phosphorylation levels of phosphatidylinositol 3-kinase (PI3K) and Akt (protein kinase B, PKB); reducing lactate dehydrogenase leakage rates and reactive oxygen species levels, and downregulating the levels of inflammatory factors such as IL-1β, IL-6, and TNF-α (Ma et al., 2023). Another study found that after administering astragaloside IV combined with MSC exosomes to rats with AMI, the infarct size, LVEDS, and LVEDD were significantly reduced, LVEF and LVFS were significantly increased, and myocardial tissue IL-6, TNF-α, and apoptosis rates were significantly reduced. Pyruvate dehydrogenase alpha (Recombinant Pyruvate dehydrogenase alpha 1, PDHA1) was identified as the key factor involved in this process (Zhongxin et al., 2025).

### 3.4 Vascular remodeling

Vascular remodeling (VR) is a process involving changes in the cell types, morphology, and function of blood vessels caused by abnormal hemodynamics. It represents a significant risk factor for the progressive development of hypertension and target organ damage (Li et al., 2024; Rizzoni et al., 2023). Ginsenoside Rg1 is a triterpene saponins with pharmacological activities such as cardiovascular protection, anti-inflammation and immune regulation (Yang et al., 2023). A study on endothelial injury-induced VR demonstrated that ginsenoside Rg1 upregulated the expression of human umbilical vein endothelial cell-derived exosomal miR-7977, decreased the expression of synthetic marker proteins osteopontin (OPN), non-muscle myosin heavy chain isoform-B (Smemb), and cellular retinol binding protein-1 (CRBP-1), while increasing the expression of contractile marker proteins α-SMA, smooth muscle myosin heavy chain (SM-MHC), and Smoothelin-B. It also targeted and inhibited the mitogen-activated protein kinase 13 (MAPK13) gene, inhibiting the phenotypic transformation and proliferation of vascular smooth muscle cells and promoting vascular repair (Shuai, 2022).

Vascular calcification (VC) is a systemic and dynamic vascular disease, which refers to the ectopic deposition of hydroxyapatite minerals in the arterial wall, often accompanied by vascular remodeling, and is closely related to cardiovascular diseases (Lee et al., 2020). Curcumin is a phenolic compound with anti-inflammatory, anti-hyperlipidemic, and antioxidant properties. Chen et al. found that curcumin reduces vascular calcification in rat aortas by downregulating the expression of transcription factor KLF4 and osteogenic factor RUXN2 through upregulating the expression of miR-92b-3p in exosomes derived from vascular smooth muscle cells (Chen et al., 2022).

### 3.5 Other mechanisms

Autophagy plays a dual role in CVDs through adaptive or maladaptive regulation. Moderate autophagy protects cardiomyocytes by clearing damaged organelles accumulated during ischemia, whereas excessive or insufficient autophagy exacerbates cardiomyocyte apoptosis and structural damage, worsening the severity of MI/RI (Mei et al., 2015). Oridonin is a terpenoid compound exhibiting a wide range of biological activities, including anti-inflammatory and antitumor effects (He et al., 2018; Zhou et al., 2023). Fu et al. found that Oridonin combined with exosomes derived from in BMSC can inhibit MIRI, which was represented by the ability of exosomes treated with oridonin to significantly reduce the number of apoptotic cardiomyocytes and reverse the increasing trend of cardiac parameters such as heart rate, left ventricular systolic pressure (LVSP), LVFS, LVEF and left ventricular wall thickness (LVWT). The expression of recombinant human beclin 1 protein (Beclin-1), autophagy-related protein 13 (ATG13), and B-cell lymphoma-2 gene (Bcl-2) was upregulated, the expression of apoptotic protease activating factor-1 (Apaf1), B-cell lymphoma-2-associated X protein (Bax) and proliferation cell nuclear antigen Ki67 was downregulated, and the proportion of edu-positive cells was increased (Fu et al., 2021).

## 4 Conclusion and outlook

**FIGURE 1:**
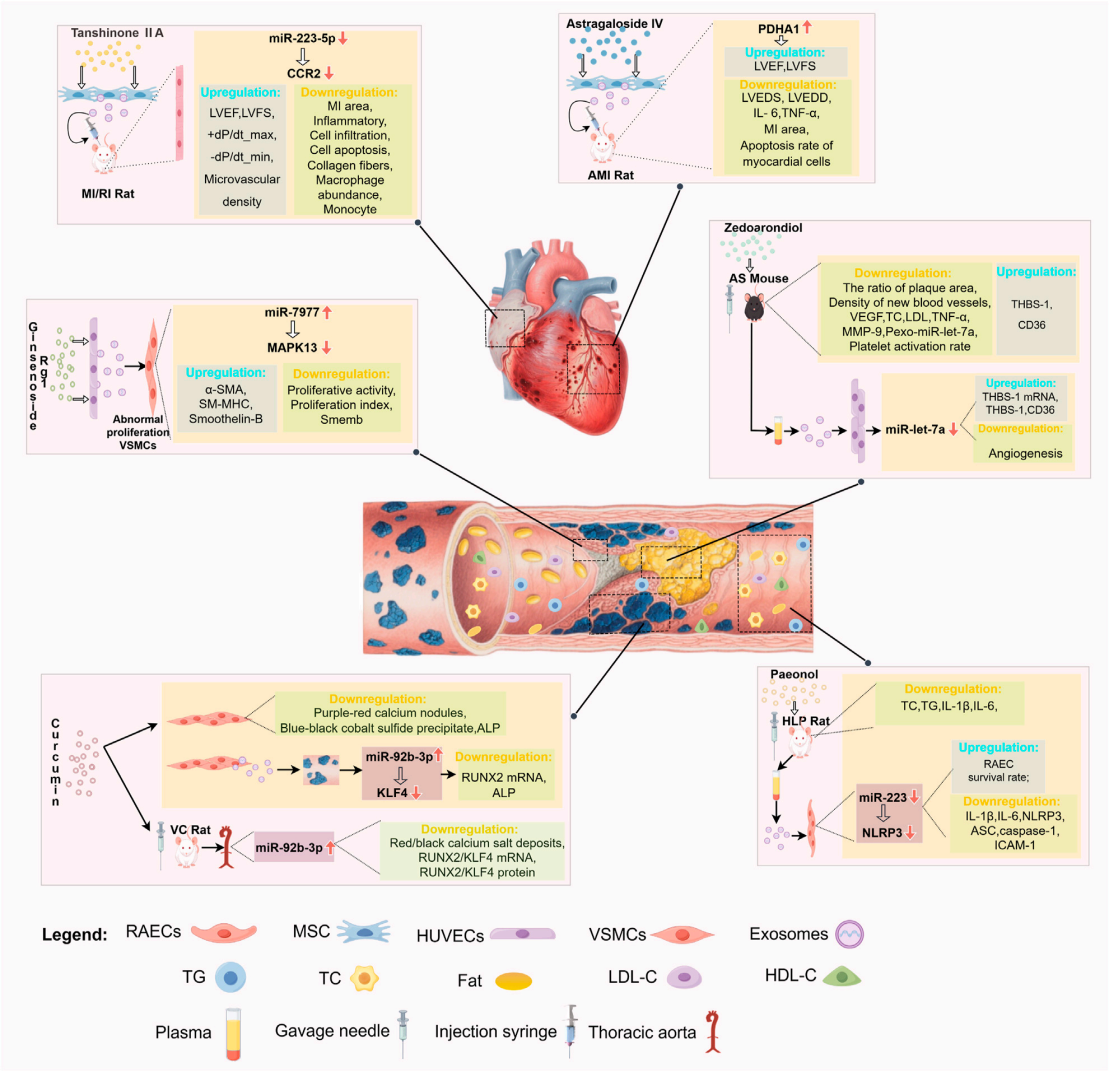
Medicinal plant-derived metabolites-exosomes miRNAs-main cardiovascular effects.

Medicinal plant-derived metabolites show remarkable potential in cardiovascular disease protection by regulating exosomal miRNAs or synergizing with exosomes, and their multi-target and multi-pathway action properties provide new strategies to overcome the limitations of traditional drugs. This study summarizes the effects of terpenoids (such as Zedoarondiol, Tanshinone ⅡA, and Oridonin), phenols (such as Paeonol and Curcumin), polysaccharides (e.g., astragalus polysaccharide), and triterpenoids (e.g., astragaloside IV, ginsenoside Rh2, and ginsenoside Rg1) play a role in promoting/inhibiting angiogenesis, anti-inflammatory and anti-oxidative stress in AS, MIRI, and AMI by regulating exosomal miRNA expression or synergizing with exosomes (Figure 1). Although these medicinal plant-derived metabolites show promise in the treatment of CVD, the existing research has certain limitations: (1) Most studies focus on a single component regulating specific miRNAs, but exosomal miRNA expression is dynamic (Yu et al., 2016). Whether these metabolites act via miRNA networks (not single molecules) needs verification with single-cell sequencing and high-throughput screening. (2) Translating basic research to clinical use faces challenges (e.g., standardized metabolite extraction, optimized drug delivery), requiring pharmaceutical/bioengineering breakthroughs to build a standardized extraction-testing system and more preclinical pharmacodynamic evaluations. (3) There are few studies on these metabolites regulating exosomal miRNA for CVD treatment, lacking basic/clinical data on diseases like hypertension, arrhythmia, heart failure, and cardiomyopathy, as well as research on other metabolites for this purpose. Expanding CVD study scope/models, exploring more metabolites’ potential, and integrating CRISPR (to identify key exosomal miRNA nodes) with a focus on clinical translation are needed. (4) Due to their small size, high heterogeneity, and susceptibility to interference from other components in biological fluids, exosome isolation methods (such as ultracentricentrifugation, magnetic bead sorting, etc.) and quantification techniques (such as nanoparticle tracking analysis, Western blot quantification, etc.) have yet to establish unified standards. It is essential to compare the principles underlying current mainstream separation and quantification methods, analyze the advantages and limitations of each approach, explore the core elements of standardized protocols, and define critical parameters to enhance the reliability and reproducibility of results. (5) Previous studies have primarily focused on the identification of plant metabolite components and their biological activities, with insufficient attention given to pharmacokinetic research, namely the absorption, distribution, metabolism, and excretion (ADME) processes within the body. Future studies should supplement these analyses by investigating their stability in the gastrointestinal tract, transmembrane transport mechanisms, hepatic metabolic pathways, and excretion routes. Integrating experimental data from animal models, such as changes in blood drug concentrations and tissue distribution patterns will elucidate how *in vivo* metabolic processes influence bioavailability and efficacy. This will also facilitate discussions on potential formulation optimization strategies to enhance pharmacokinetic performance. (6) Included literature relies heavily on *in vitro* experiments and animal models, lacking clinical sample verification. Future research should emphasize clinical sample collection/analysis and validation to confirm these metabolites’ effectiveness/safety in regulating exosomal miRNA for CVD protection.

In summary, research on the protection of CVD using medicinal plant-derived metabolites via exosome-derived miRNAs or synergistic exosome pathways holds both fundamental theoretical value and clinical translation potential. With advancements in research methods and technological progress, such medicinal plant-derived metabolites are poised to become a key source for the next-generation of CVD therapies, offering safer and more effective solutions to global cardiovascular health challenges.
